# Comparison of Corneal Endothelial Imaging Techniques by Specular Microscopy in Unsedated Healthy Dogs

**DOI:** 10.1111/vop.70182

**Published:** 2026-04-17

**Authors:** Hyunwoo Suk, Minju Kim, Dohyeong Kim, Seonmi Kang, Kangmoon Seo

**Affiliations:** ^1^ Department of Veterinary Clinical Sciences, College of Veterinary Medicine and Research Institute for Veterinary Science Seoul National University Seoul Korea

**Keywords:** canine, corneal endothelial dystrophy, corneal endothelium, specular microscopy, specular reflection, unsedated

## Abstract

**Objective:**

To compare unsedated noncontact specular microscopy imaging techniques for the canine corneal endothelium and identify the most effective technique.

**Animals Studied:**

Nineteen eyes of 10 systemically healthy, staff‐owned dogs with clinically normal corneas were studied.

**Procedure:**

Six imaging groups were divided according to the different techniques used in all eyes: central focusing, with (CP) or without (CN) induction of an indirect pupillary light reflex (PLR); peripheral focusing with (PP) or without (PN) an indirect PLR; PP with application of a contact lens (CL); and gaze fixation with a treat (GF). The success rates, imaging times, and endothelial cell indices were compared.

**Results:**

Success rates differed significantly among the techniques, with PP and CL achieving significantly higher success rates than CN. When only successful images were analyzed, the mean imaging time showed no statistically significant differences between the groups. In the sensitivity analysis, PP required significantly shorter imaging times than CN and PN. The Endothelial cell indices (cell count, endothelial density, and hexagonality) did not differ significantly among the techniques.

**Conclusions:**

Peripheral focusing combined with the induction of an indirect PLR (PP) achieved significantly higher success rates and shorter imaging times than other techniques, suggesting that this technique provides the most reliable and efficient approach for evaluating the canine corneal endothelium with noncontact specular microscopy under unsedated conditions.

## 1 Introduction

The corneal endothelium is the innermost layer of the cornea and is composed of a monolayer of hexagonally arranged cells [1]. The corneal endothelium contributes to the preservation of corneal transparency by sustaining dehydration through the combined action of its barrier properties and the activity of Na^+^/K^+^‐ATPase pumps [2]. Normal corneal endothelial cells tend to decrease in density and enlarge in size with advancing age in dogs [3]. In younger dogs, the endothelial cell density (ECD) is reported to be approximately 3000 cells/mm^2^ [4]. Because the canine corneal endothelium has limited proliferative capacity, damage results in a reduction of ECD [5]. When ECD decreases below 500–800 cells/mm^2^, the endothelium can no longer remove fluid from the stroma, resulting in corneal edema [6]. Endothelial cells may be affected by various factors, including genetic predispositions such as corneal endothelial dystrophy, exogenous trauma such as anterior lens luxation, corneal or intraocular surgery, and intraocular inflammation such as anterior uveitis and glaucoma [5, 7, 8, 9]. Since these parameters directly influence the cornea's capacity to maintain transparency and are critical in predicting clinical outcomes in conditions such as endothelial dystrophy or after intraocular surgery, evaluating the number and morphology of corneal endothelial cells is of great clinical importance.

Specular microscopy allows the morphological analysis of corneal endothelial cells using a non‐invasive technique. It operates on the principle of specular reflection, in which projected light is reflected at an equal angle, and approximately 0.022% of the projected light is reflected at the interface between the endothelium and aqueous humor to form an image [10]. Clinical use of specular microscopy can be categorized into the contact and noncontact types. Contact specular microscopes require applanation by placing the objective lens directly on the corneal surface, whereas noncontact instruments employ automated focusing technology to image the endothelium. In human ophthalmology, specular microscopy is widely used for the diagnosis of primary corneal endothelial disorders, such as Fuch's endothelial corneal dystrophy and congenital hereditary endothelial dystrophy, as well as for the evaluation of surgical outcomes following intraocular surgeries (e.g., phacoemulsification), and assessment of endothelial changes associated with contact lens wear and other secondary corneal endothelial disorders [11].

In veterinary ophthalmology, the use of specular microscopy presents challenges compared with human medicine. In dogs, reflection from the tapetum lucidum can interfere with image acquisition, making it more difficult to obtain clear corneal endothelial images. As a result, imaging is often technically demanding and may require sedation or anesthesia. Prior studies have been conducted under sedation, general anesthesia, or using enucleated eyes [3, 12, 13, 14, 15, 16]. Sedation, general anesthesia, or enucleated conditions may alter the physiological state of the cornea and present limitations for repeated application in clinical practice. These limitations highlighted the need to identify practical imaging strategies that facilitated reliable specular microscopy in unsedated canine patients. Thus, the aim of this study was to evaluate the feasibility of performing specular microscopy using various imaging techniques in dogs without sedation and to determine the best optimized technique for clinical application.

## 2 Materials and Methods

### 2.1 Animals

This study was conducted on 10 staff‐owned dogs at Seoul National University Veterinary Medical Teaching Hospital. The dogs included in this study were systemically healthy, had no detectable corneal abnormalities on slit‐lamp biomicroscopy or basic ophthalmic examinations, and had clinically normal corneas. There were no restrictions on breed, age, sex, or neuter status. This study was approved by the Institutional Animal Care and Use Committee of Seoul National University (SNU‐250818‐3).

### 2.2 Ophthalmic Examination

All dogs underwent routine ophthalmic examinations before endothelial imaging. Basic ophthalmic examinations including menace response, dazzle reflex, direct/indirect pupillary light reflex (PLR), Schirmer tear test (STT‐1), and intraocular pressure measurement using rebound tonometry (TonoVet, iCare, Finland) were performed. Slit‐lamp biomicroscopy (Topcon Model SLD7, Topcon Corp.) was then performed to evaluate for corneal abnormalities such as ulceration, deposits, edema, or pigmentation that could interfere with endothelial imaging. Eyes with corneal abnormalities were excluded from the study. In addition, fundus photographs were obtained to document the posterior segment status.

### 2.3 Specular Microscopy

**FIGURE 1:**
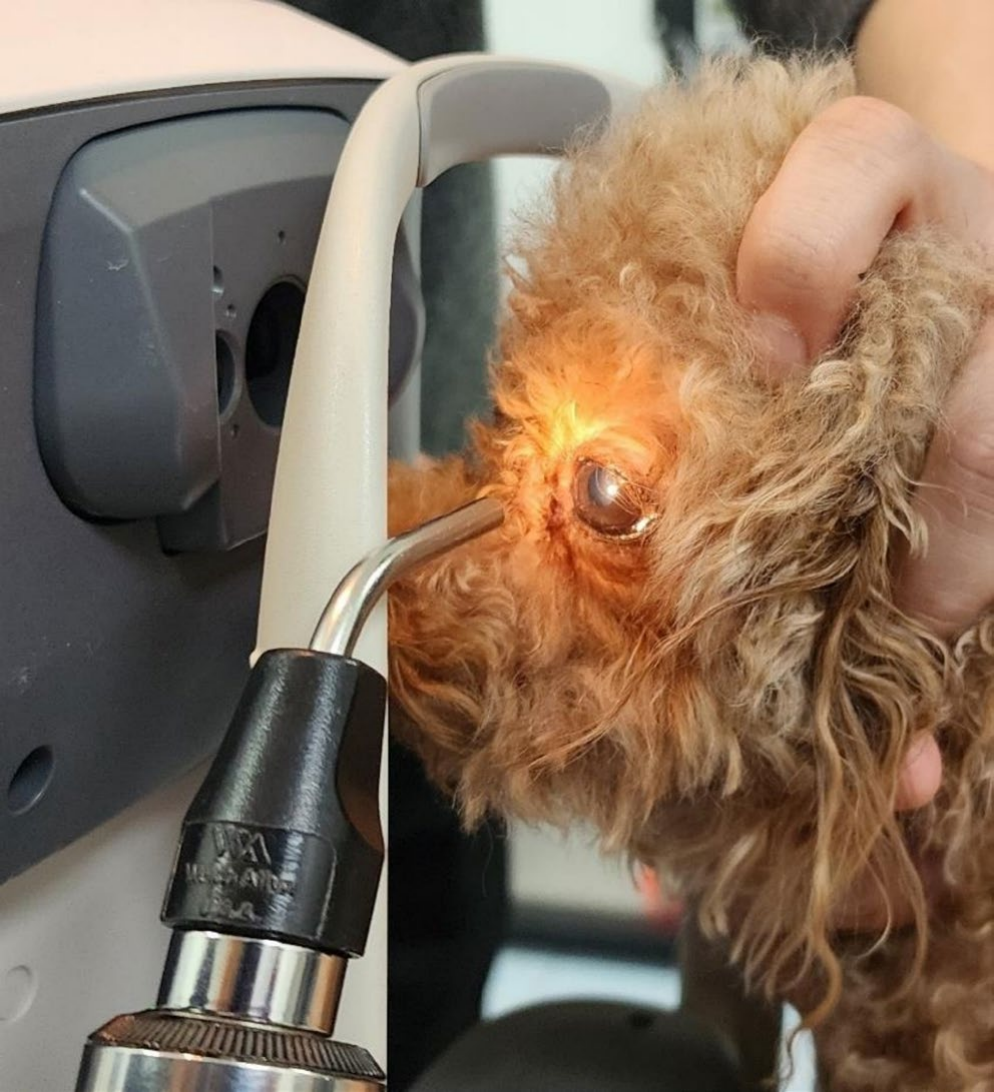
Representative photograph demonstrating the image acquisition set up for laterally focused, inducing miosis with indirect pupillary light reflex (PLR) in the right eye (PP technique). One handler stabilized the dog's head, while another handler restrained the body to minimize movement. An assistant applied a light stimulus to the left eye to induce an indirect PLR during image acquisition. PP, lateral corneal focus after inducing miosis by indirect PLR.

The experiments were conducted under indoor lighting conditions. One examiner restrained the body of each dog, while another stabilized the head, with the same individual consistently performing head restraint throughout the study (Figure 1).

Corneal endothelial imaging was performed using a noncontact specular microscopy (EM‐4000, Tomey Corp.). Each eye was examined using six different imaging technique; focus placed on the central cornea without any additional procedure (Group CN), focus placed 3‐4 mm lateral to the central cornea without any additional procedure (Group PN), focus placed on the central cornea after inducing miosis by indirect PLR (Group CP), focus placed 3‐4 mm lateral to the central cornea after inducing miosis by indirect PLR (Group PP), application of PP while a contact lens (PureVision, Bausch & Lomb) was fitted (Group CL), and application of PP while the dog's gaze was directed toward identical treats from the same commercial product, positioned approximately 10 cm from the nose (Group GF). The order of application was randomized using an online randomization program (www.random.org). Each technique was repeated three times.

To minimize corneal surface drying from repeated imaging, 0.3% hyaluronate artificial tears were instilled after three trials, 2–3 min before proceeding to the next imaging technique.

Imaging time was measured after ocular alignment, from initiation of automatic focusing until a “complete” message appeared on the screen.

**FIGURE 2:**
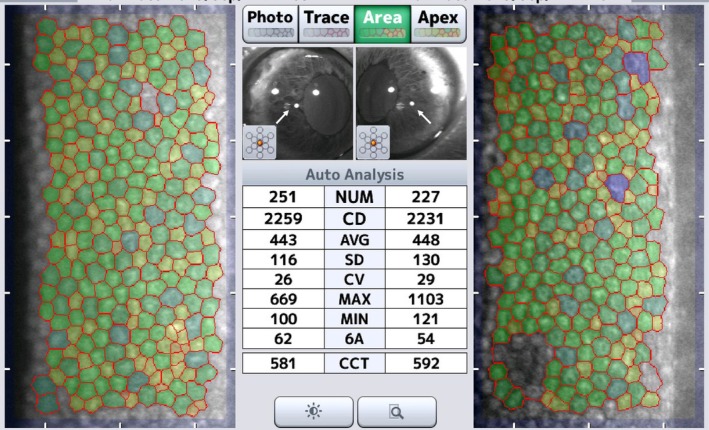
Representative corneal endothelial image obtained in group PP. This image was acquired by combining peripheral focusing with induction of an indirect pupillary light reflex (PLR) (white arrow). Distinct cell borders and sufficient cell counts for morphometric analysis were visible. Only the cells highlighted in color (yellow/green/purple) were included in the analysis and counted.

“Success” was defined as the acquisition of images in which at least 150 endothelial cells were clearly captured, enabling the calculation of ECD and hexagonality (6A) using the built‐in automated analysis software (Figure 2).

“Failure” was defined as if the procedure exceeded 30 s or if fewer than 150 endothelial cells were captured.

### 2.4 Statistical Analyses

A generalized estimating equation (GEE) logistic regression with an exchangeable correlation structure was used to evaluate the differences in imaging success between techniques and between eyes, with EMMs and 95% Wald CIs. Pairwise comparisons were performed using Bonferroni correction for multiple testing.

Imaging time was analyzed using GEE linear regression (normal distribution, identity link) with technique, eye, and trial number as covariates. A sensitivity analysis was performed by imputing failed attempts at 35 s.

For cell count, ECD, and hexagonality, a linear mixed model was applied with technique, eye, and trial number as fixed factors. Estimated marginal means (EMMs) with 95% confidence intervals (CIs) were obtained, and pairwise comparisons were adjusted using Bonferroni correction.

All statistical analyses were performed using SPSS. Statistical significance was set at *p* < 0.05.

## 3 Results

### 3.1 Animals

A total of 10 dogs were enrolled in the study and labeled D01‐D10. The study population comprised a mixed‐breed (*n* = 4), Poodles (*n* = 2), Cavalier King Charles Spaniel (*n* = 1), Chihuahua (*n* = 1), Pomeranian (*n* = 1), and Maltese (*n* = 1). There were seven spayed females and three castrated males with a mean age of 9.4 ± 3.1 years (range: 6–15 years) (Table 1).

**TABLE 1:** Demographic of dogs included in the study.

Dog ID	Age	Breed	Sex	Ocular abnormalities
D01	6	Mixed	FS	—
D02	8	Mixed	FS	OU: incipient cataract
D03	7	CKCS	FS	—
D04	7	Chihuahua	MC	OS: incipient cataract
D05	11	Poodle	MC	OU: incipient cataract
D06	15	Poodle	FS	OU: incipient cataract
D07	10	Pomeranian	FS	OD: incipient cataract, OS: upper eyelid mass
D08	14	Mixed	FS	OU: incipient cataract
D09	9	Maltese	MC	OS: incipient cataract, corneal edema^a^
D10	7	Mixed	FS	—

Abbreviations: CKCS, Cavalier King Charles Spaniel; FS, spayed female; MC, castrated male; OD, right eye; OS, left eye; OU, both eye.

^a^ Left eye of D09 was excluded from analysis due to corneal edema.

### 3.2 Ophthalmic Examination

A total of 19 eyes were included in the study. One eye was excluded from the analysis due to corneal edema (D09 OS). Tear production was within normal range in all eyes. Intraocular pressure was also within the normal range. All dogs exhibited bilateral iris atrophy; and D05 and D06 were severe, resulting in minimal pupillary constriction. Focal cataracts were observed in 11 eyes, and one eye (D07 OS) exhibited an upper eyelid mass. No corneal abnormalities were detected in any of the eyes (Table 1).

### 3.3 Specular Microscopy

#### 3.3.1 Success Rate

From the 342 imaging trials, 124 trials were successful. The number of successful image acquisitions in each group was as follows: CN (9/57), PN (13/57), CP (26/57), PP (28/57), CL (26/57) and GF (22/57).

A GEE logistic regression analysis revealed a significant effect of imaging technique on success (Wald χ^2^ = 18.997, df = 5, *p* = 0.002). The EMMs of the predicted success rates were 16% (95% CI: 5–39), 23% (95% CI: 9–46), 46% (95% CI: 27–66), 50% (95% CI: 36–63), 46% (95% CI: 33–60), and 39% (95% CI: 24–56) for groups CN, PN, CP, PP, CL, and GF, respectively (Table 2).

**TABLE 2:** Number of successful acquisitions, estimated marginal means of imaging success and endothelial cell indices in this study.

Groups	Number of success aquisition (n)	Success rate (%, 95% CI)	Endothelial cell indices		
			Cell count (n)	ECD (cells/mm^2^)	6A (%)
CN	9	16 (5–39)	231.8 ± 10.1	2288.6 ± 53.8	59.2 ± 2.4
PN	13	23 (9–46)	231.5 ± 8.4	2274.8 ± 44.9	58.4 ± 2.0
CP	26	46 (27–66)	212.9 ± 6.0	2233.0 ± 31.8	55.1 ± 1.4
PP	28	50 (36–63)	211.2 ± 5.9	2245.7 ± 31.2	58.9 ± 1.4
CL	26	46 (33–60)	212.4 ± 6.0	2247.9 ± 31.7	55.6 ± 1.4
GF	22	39 (24–56)	220.9 ± 6.5	2222.6 ± 34.6	58.1 ± 1.5

Abbreviations: 6A, Hexagonality; CI, Confidence Interval; CL, PP method with a fitted contact lens; CN, central corneal focus without any additional procedure; CP, central corneal focus after inducing miosis by indirect pupillary light reflex (PLR); ECD, Endothelial Cell Density; GF, PP method with visual fixation using a treat positioned approximately 10 cm from the nose; PN, lateral corneal focus without any additional procedure; PP, lateral corneal focus after inducing miosis by indirect PLR.

**FIGURE 3:**
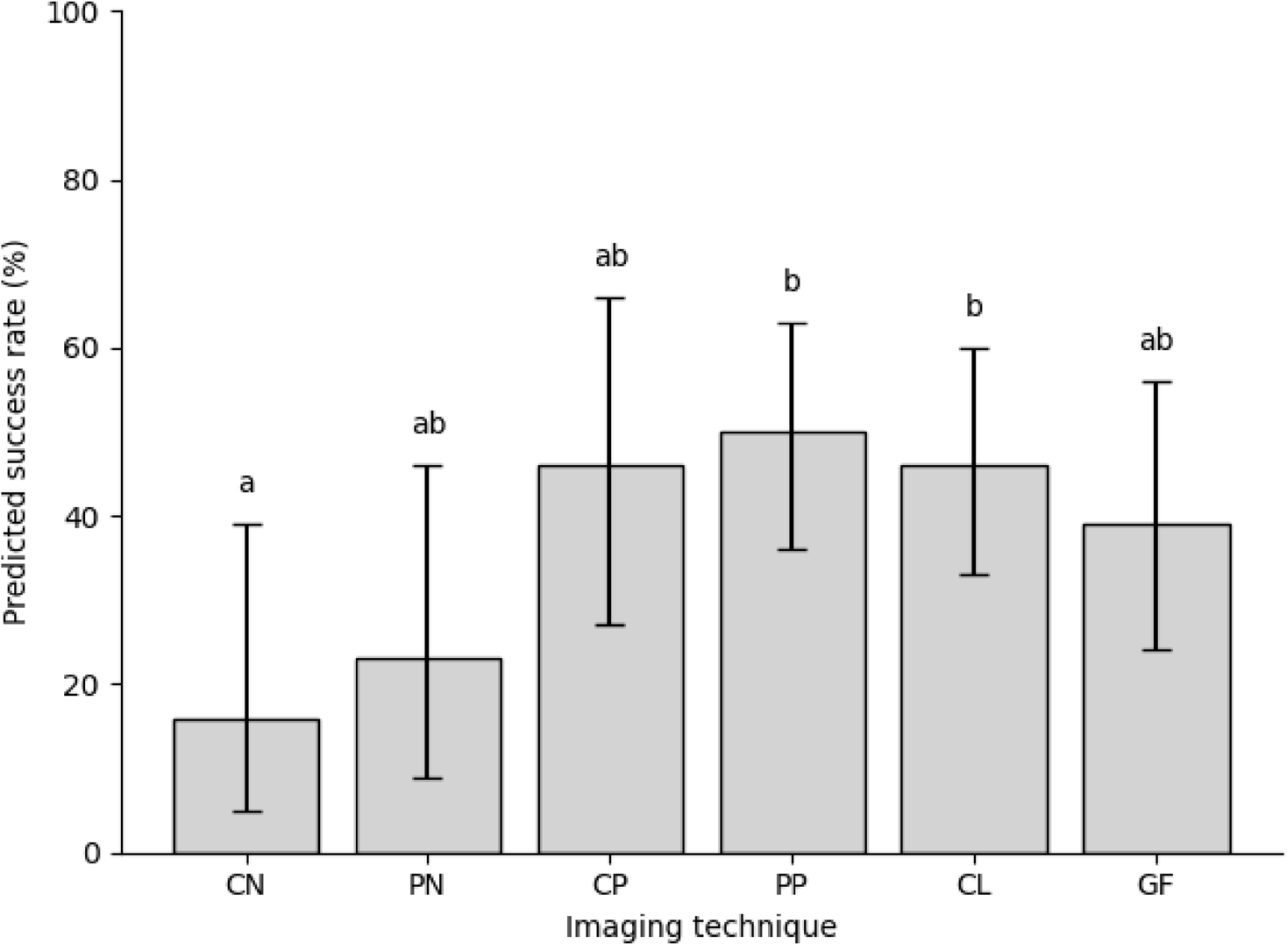
Predicted imaging success rates by method with 95% confidence intervals (CI). Bars represented the estimated marginal means of predicted success rates. Lines represent CI. Different superscripts above the 95% CI (a, b) indicate statistically significant differences among groups (*p* < 0.05, pairwise comparisons with Bonferroni correction). CN, central corneal focus without any additional procedure; PN, lateral corneal focus without any additional procedure; CP, central corneal focus after inducing miosis by indirect pupillary light reflex (PLR); PP, lateral corneal focus after inducing miosis by indirect PLR; CL, PP method with a fitted contact lens; GF, PP method with visual fixation using a treat positioned approximately 10 cm from the nose.

Pairwise comparisons revealed that groups CP, PP, and CL achieved significantly higher success rates than groups CN and PN (CP–CN, *p* = 0.007; PP–CN, p < 0.001; CL–CN, *p* = 0.002; CP–PN, *p* = 0.008; PP–PN, *p* = 0.008; CL–PN, *p* = 0.042). However, after Bonferroni correction for multiple testing, only comparisons between PP and CN (*p* = 0.012) and between CL and CN (*p* = 0.035) remained statistically significant. No other pairwise differences were observed between the groups after correction (*p* > 0.05) (Figure 3).

#### 3.3.2 Imaging Time

When only “success” images were analyzed, the overall mean imaging time was 9.85 ± 6.21 s. The predicted mean times (seconds, SD) for each group were 8.92 ± 2.10, 11.91 ± 1.17, 12.39 ± 2.03, 9.16 ± 1.54, 9.90 ± 1.03, and 9.13 ± 1.08 in groups CN, PN, CP, PP, CL, and GF, respectively. GEE analysis demonstrated a significant overall effect of technique (Wald χ^2^ = 24.664, df = 5, *p* < 0.001). Pairwise comparisons indicated that PN required significantly longer times than PP (*p* = 0.006) and CP required significantly longer times than GF (*p* = 0.020) before adjustment. However, after Bonferroni correction for multiple comparisons, no statistically significant differences were observed between the individual groups (*p* > 0.05).

**FIGURE 4:**
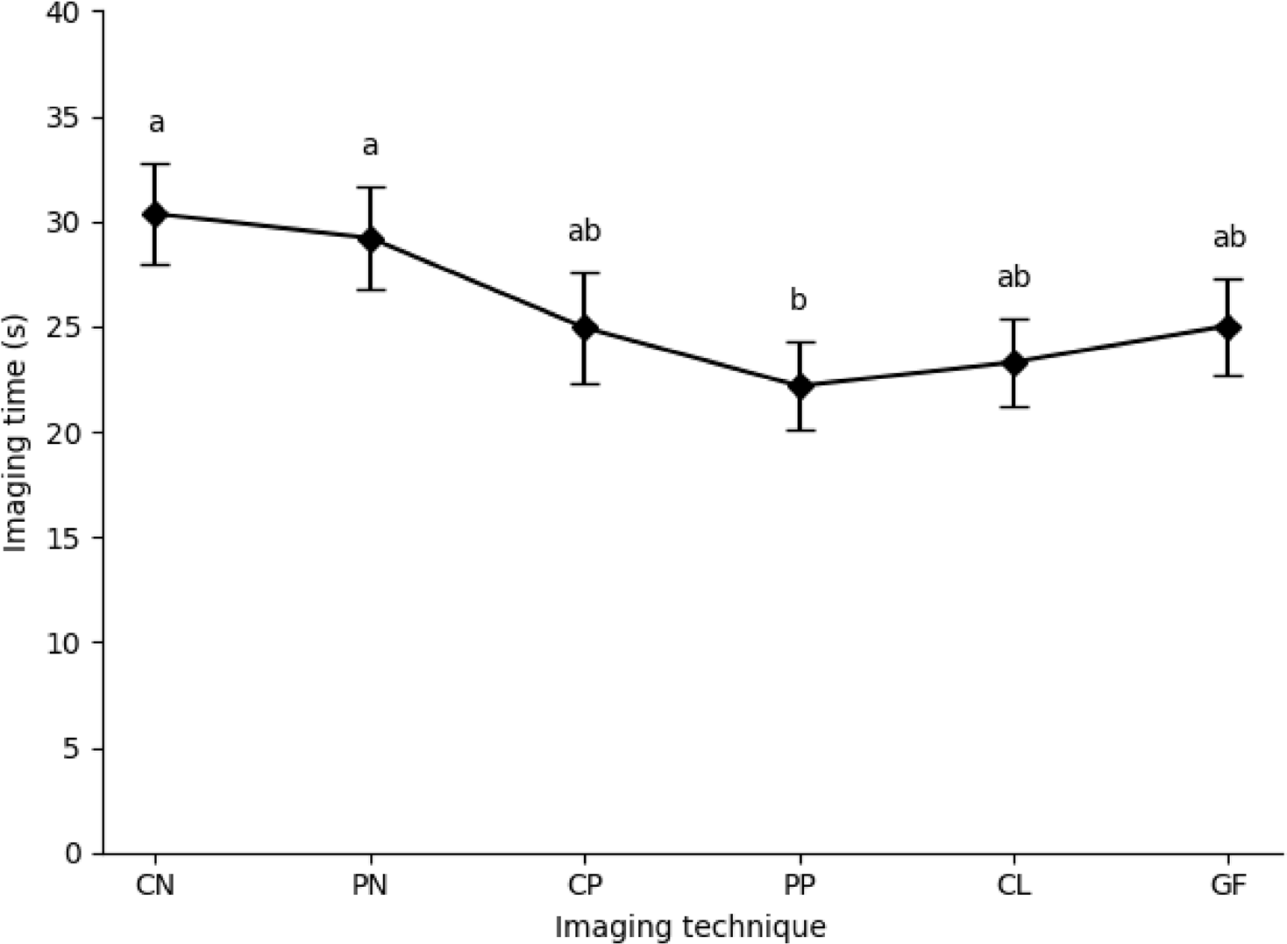
Imaging times by groups with 95% confidence intervals. Black squares represents mean times when failed attempts were imputed as 35 s. Vertical bars denote 95% CI. Different superscripts (a, b) above the bars indicated statistically significant differences between methods after Bonferroni correction (*p* < 0.05). CN, central corneal focus without any additional procedure; PN, lateral corneal focus without any additional procedure; CP, central corneal focus after inducing miosis by indirect pupillary light reflex (PLR); PP, lateral corneal focus after inducing miosis by indirect PLR; CL, PP method with a fitted contact lens; GF, PP method with visual fixation using a treat positioned approximately 10 cm from the nose.

When failed attempts were imputed as 35 s, the overall mean imaging time increased to 25.96 ± 12.64 s. The predicted mean times (seconds, SD) for each group were 30.37 ± 2.39, 29.22 ± 2.47, 24.95 ± 2.62, 22.18 ± 2.07, 23.31 ± 2.09, and 25.04 ± 2.29 in groups CN, PN, CP, PP, CL, and GF, respectively. GEE analysis again demonstrated a significant overall effect of technique (Wald χ^2^ = 19.925, df = 5, *p* = 0.001). In pairwise comparisons, CN and PN required significantly longer times than CP, PP, and CL (*p* < 0.05). However, after Bonferroni correction, only the differences between CN and PP (*p* = 0.019) and PN and PP (*p* = 0.021) remained statistically significant (Figure 4). In addition, Trial number had no significant effect (Wald χ^2^ = 1.132, df = 2, *p* = 0.568).

When only “success” images were analyzed, right eyes required 10.9 ± 0.77 s and left eyes required 9.2 ± 0.87 s, with a statistically significant difference (*p* = 0.003). However, when failed attempts were imputed as 35 s, right eyes required 26.1 ± 0.95 s and left eyes required 25.3 ± 1.51 s, with no significant difference between the right and left eyes (*p* = 0.616).

#### 3.3.3 Endothelial Cell Analysis

In a total of 124 “success” images, more than 150 endothelial cells were imaged in each case, allowing automated analysis by the device; only the cells highlighted in color (yellow/green/purple) were included in the analysis and counted. The mean number of analyzed cells, ECD, and hexagonality (6A) were evaluated for each group (Table 2). The overall mean cell count was 217.2 ± 30.5, the mean ECD was 2245.9 ± 249.1 cells/mm^2^, and the mean hexagonality was 57.1% ± 7.3%.

Analysis of image quality indices revealed no significant differences among the imaging techniques in terms of cell count, ECD, or hexagonality. The mean number of cells analyzed per image ranged from 211.2 ± 5.9 to 231.8 ± 10.1, the mean ECD ranged from 2222.6 ± 34.6 to 2288.6 ± 53.8 cells/mm^2^, and hexagonality ranged from 55.1% ± 1.4% to 59.2% ± 2.4%. Statistical analysis confirmed that none of these parameters differed significantly between the groups (*p* > 0.05).

## 4 Discussion

In this study, noncontact specular microscopy imaging was performed in 19 eyes from 10 dogs without the use of sedation or anesthesia. In some cases, proper focus could not be achieved, resulting in imaging failure. This limitation, unlike in humans, is caused in dogs by fundus reflex interference due to the presence of the tapetum lucidum, which occupies approximately 30% of the superior fundus [17]. To minimize this interference, we employed alternative strategies such as shifting the focus 3‐4 mm laterally from the central cornea or inducing miosis through indirect PLR. In one study, pharmacologic miosis with latanoprost has been used. In humans, the assessment of the corneal endothelium is based on images that include at least 150 clearly visualized cells. Consistent with this approach, the present study defined a “success” as an image containing more than 150 endothelial cells [18].

Our results demonstrated that group PP, which combined peripheral focusing with the induction of indirect PLR, achieved the highest success rate, whereas group CN showed the lowest success rate. In several dogs (D01, D02, D09, and D10), imaging could not be achieved with the CN or PN technique because of interference from tapetal reflection. However, endothelial imaging became possible after miosis induction. D03 was successfully imaged using the CN technique. This was likely because the area occupied by the tapetum lucidum was relatively small, resulting in minimal tapetal reflection interference. D07, which had relatively mild iris atrophy, could also be imaged using the CN technique. D05, who presented with severe iris atrophy, could not be imaged using any technique, whereas D06, despite showing a similar degree of iris atrophy, was successfully imaged using the PP technique. This discrepancy was likely explained by the progressed cataract and nuclear sclerosis in D06, which reduced tapetal reflection. Collectively, these findings highlighted the critical role of reducing tapetal reflection in determining the success of specular microscopy in unsedated dogs.

Group CL, in which contact lenses were applied, with the intention of creating a more uniform and optically smooth corneal surface, which was expected to facilitate specular reflection and improve image acquisition, demonstrated a statistically higher success rate; however, its absolute likelihood of success was lower than that of the group PP. Therefore, their clinical advantages would be limited. This limitation might be attributable to individual variation in corneal curvature among dogs, as well as the possibility of lens deformation when manipulating the periocular region during the procedure. Meanwhile, although group CP did not show a statistically significant improvement, it achieved a higher success rate than groups CN and PN, suggesting that in clinical practice, the CP technique alone might still provide a reasonable chance for successful imaging. Group GF, which involved presenting a treat to induce gaze fixation under unsedated conditions, also showed a relatively higher success rate; however, the difference was not statistically significant. This outcome was likely attributable to variations in treat preferences among individual dogs and agitation elicited in some cases. Future studies would be warranted to develop and validate more stable and effective gaze fixation techniques that could be applied in clinical settings as alternatives to the GF technique.

In the analysis of imaging time, group CN showed the shortest duration when only successful trials were considered; however, the small number of successful attempts limited its statistical reliability. In the sensitivity analysis, in which failed attempts were imputed as 35 s, group PP demonstrated the shortest imaging time, whereas group CN required the longest time. These findings indicated that group PP consistently exhibited greater efficiency in both analyses. The imputation of failed attempts as 35 s was performed because analyzing only successful trials would have reduced the statistical reliability of the comparison for some imaging techniques with relatively low success rates. By assigning a fixed duration of 35 s to unsuccessful attempts, the analysis was designed to reflect not only the time required for successful image acquisition but also the frequency of failure, thereby providing a more realistic assessment of overall imaging efficiency across techniques.

The observed difference in imaging time between the left and right eyes in the initial analysis was likely influenced by the low number of successful trials for some imaging techniques, which might have reduced the statistical reliability of the comparison when only successful attempts were analyzed. When a sensitivity analysis was performed in which failed attempts were imputed as 35 s, no statistically significant difference in imaging time was found between the two eyes. This suggested that the apparent inter‐eye difference observed in the analysis of successful trials alone might not be statistically robust.

When both imaging success and examination time were evaluated, group PP consistently demonstrated superior performance compared with the other approaches. By focusing on the peripheral cornea, this approach avoided the central region where interference from tapetal reflection is most pronounced in dogs, thereby reducing light from tapetum. In addition, indirect pupillary light reflex (PLR) induced miosis, allowing the iris to partially block light reflected from the tapetum lucidum and thereby minimizing interference during image acquisition. Together, these factors likely contributed to the higher success rate and shorter imaging time observed in group PP. These findings suggested that the PP technique not only maximized the likelihood of obtaining analyzable endothelial images but also enhanced clinical efficiency, thereby making it the most practical and reliable technique for corneal endothelial assessment under unsedated conditions.

In the analysis of endothelial cell indices, the overall mean ECD of this study was 2245 cells/mm^2^. In prior studies using specular microscopy, one study examined dogs between 2 and 11 years of age and reported a mean ECD of approximately 2200 cells/mm^2^, which is comparable to the present findings [5]. Two other studies investigated 6–9 months dogs, and mean age of 6 years reported mean ECD of approximately 2500 cells/mm^2^, values somewhat higher than those obtained in this study [12, 16]. Such differences were most likely explained by differences in the age distribution of the study populations. Indeed, prior study reported that dogs younger than 1 year exhibited ECD exceeding 2600 cells/mm^2^, which decreased to 2300–2500 cells/mm^2^ in dogs aged 1–9 years, and further declined to 1900–2100 cells/mm^2^ in dogs older than 10 years [3]. Considering these age‐related patterns, the mean ECD observed in our study could be considered consistent with values reported in prior studies.

The overall mean hexagonality in this study was 57.1%, which was lower than the 68%–69% reported in an ex vivo study [15]. This difference was likely attributed to the disparity between in vivo imaging under unsedated conditions and ex vivo experimental settings. In humans, it is generally accepted that more than 60% of corneal endothelial cells exhibit a hexagonal shape [19].

In addition, no significant differences were found between the imaging techniques in terms of cell count, ECD, and hexagonality (*p > 0.05*). This suggests that once image acquisition was successful, the derived morphometric parameters were comparable across groups, which is reasonable given that the same corneal endothelium was imaged.

However, this study had several limitations. First, the number of animals was limited, which restricted the ability to account for individual factors such as breed and age. Therefore, future studies including a larger number of dogs would be warranted to better evaluate the influence of these variables. Second, the device was originally designed for human use and the chin and forehead rests occasionally interfered with proper positioning depending on the cranial conformation of the dogs. In one study using confocal microscopy, these supports were removed to facilitate imaging and similarly modifying or removing such components may improve image quality in canine patients [4]. Another limitation of this study was that the endothelial cell indices were calculated using the automated analysis provided by the instrument. Although automated analysis offers advantages in terms of convenience and reproducibility, its accuracy can be affected by image quality or errors in cell border recognition, leading to potential bias compared to manual correction by an experienced examiner. Similar limitations of automated analysis have been reported in human studies [20], and prior studies in dogs have also highlighted these concerns [21]. Therefore, future investigations should consider combining automated analysis with manual correction or implementing more sophisticated algorithms to improve the reliability of the results.

Although the present study was conducted using a single device, the imaging strategies and considerations described here would be expected to be applicable to other specular microscopy systems that relied on specular reflection. However, endothelial cell measurements obtained using different noncontact specular microscopes are not always directly comparable in human studies, highlighting the potential influence of device‐related factors on measured outcomes [22]. Accordingly, further studies directly comparing multiple specular microscopy systems in canine patients would be valuable to confirm the repeatability of our findings.

In previous study, specular microscopy was attempted in dogs without sedation. However, practical limitations hindered its clinical applicability and reproducibility for repeated examinations [23]. The present study had important clinical significance as it was the first to demonstrate the feasibility of corneal endothelial imaging in dogs without the use of sedation or anesthesia. This approach might be particularly valuable in patients in which sedation or anesthesia would be contraindicated due to systemic disease or advanced age and the relatively short examination time made it suitable for repeated application in clinical practice. Furthermore, as demonstrated in this study, the PP technique, which combined peripheral focusing with the induction of an indirect PLR, showed higher success rates and greater efficiency than the other techniques, suggesting that it was the most practical approach under unsedated conditions.

Further studies should investigate how much tapetal reflection should be reduced to enable successful imaging, identify the most effective approach for dogs with severe iris atrophy, and develop techniques to improve the success rate of central corneal imaging. In addition, the present study was performed in clinically normal dog corneas; future studies would be warranted to evaluate the feasibility and performance of these imaging techniques in dogs with corneal endothelial diseases, such as corneal endothelial dystrophy or corneal edema.

## 5 Conclusion

This study compared several imaging techniques for corneal endothelial evaluation using noncontact specular microscopy in unsedated dogs. Peripheral focusing with indirect pupillary light reflex (PP) showed the highest imaging efficiency, suggesting that this technique would be a practical option for obtaining reliable endothelial images in clinical veterinary settings without sedation.

## Author Contributions

**Hyunwoo Suk:** investigation, methodology, visualization, writing – original draft, software, data curation. **Seonmi Kang:** methodology, validation, writing – review and editing, supervision. **Dohyeong Kim:** methodology, investigation, software. **Kangmoon Seo:** conceptualization, funding acquisition, validation, writing – review and editing, supervision. **Minju Kim:** methodology, investigation, software.

## Ethics Statement

This study was approved by the Institutional Animal Care and Use Committee of Seoul National University (SNU‐250818‐3).

## Conflicts of Interest

The authors declare no conflicts of interest.

## Data Availability

All data generated or analyzed during this study are included in this published article.
